# Multifunctional Cyanine-Based Theranostic Probe for Cancer Imaging and Therapy

**DOI:** 10.3390/ijms222212214

**Published:** 2021-11-11

**Authors:** Cheng-Liang Peng, Ying-Hsia Shih, Ping-Fang Chiang, Chun-Tang Chen, Ming-Cheng Chang

**Affiliations:** Isotope Application Division, Institute of Nuclear Energy Research, P.O. Box 3-27, Longtan, Taoyuan 32526, Taiwan; ShihY@iner.gov.tw (Y.-H.S.); ckdopamine@iner.gov.tw (P.-F.C.); ctchen@iner.gov.tw (C.-T.C.); mcchang@iner.gov.tw (M.-C.C.)

**Keywords:** multi-modality, cyanine dye, near-infrared fluorescence imaging, nuclear imaging, photothermal therapy, targeted radionuclide therapy

## Abstract

Cancer is one of the leading causes of death in the world. A cancer-targeted multifunctional probe labeled with the radionuclide has been developed to provide multi-modalities for NIR fluorescence and nuclear imaging (PET, SPECT), for photothermal therapy (PTT), and targeted radionuclide therapy of cancer. In this study, synthesis, characterization, in vitro, and in vivo biological evaluation of the cyanine-based probe (DOTA-NIR790) were demonstrated. The use of cyanine dyes for the selective accumulation of cancer cells were used to achieve the characteristics of tumor markers. Therefore, all kinds of organ tumors can be targeted for diagnosis and treatment. The DOTA-NIR790 labeled with lutetium-111 could detect original or metastatic tumors by using SPECT imaging and quantify tumor accumulation. The β-emission of ^177^Lu-DOTA-NIR790 can be used for targeted radionuclide therapy of tumors. The DOTA-NIR790 enabled imaging by NIR fluorescence and by nuclear imaging (SPECT) to monitor in real-time the tumor accumulation and the situation of cancer therapy, and to guide the surgery or the photothermal therapy of the tumor. The radionuclide-labeled heptamethine cyanine based probe (DOTA-NIR790) offers multifunctional modalities for imaging and therapies of cancer.

## 1. Introduction

Early cancer detection represents the most promising way to reduce the growth of cancer [1]. Additionally, about 1000 million people worldwide suffer from cancer every year, with an increase number of people with cancer around the world, the development of drugs for cancer diagnosis and treatment is an important part of the biological medicine. The most common form of cancer is a solid tumor of the lung, breast, prostate, colon, and rectum. If the cancer can be diagnosed at an early stage, most cancer patients after surgery, radiotherapy, chemotherapy, or combination therapy can survival for a long time. The five-year survival rate of for early-stage cancer is up to 90%.

Therefore, accurate early detection makes it possible to perform all kinds of treatment prior to tumor progression. The cancer currently can be detected by many imaging modalities, including computed tomography (CT), single-photon emission computed tomography (SPECT), positron emission tomography (PET), ultrasonography, and magnetic resonance imaging (MRI) [2]. However, there are still many significant challenges in using molecular imaging for cancer detection, including that most of the specific receptors are not overexpressed in all types of cancer. Therefore, the development of a versatile molecular probe to detect the majority of cancer tissue has considerable benefits.

Near infrared fluorescent heptamethine cyanine dyes including indocyanine green (ICG), IR-783 and IR-780 iodide, has been shown to have abilities for noninvasive tumor imaging [3,4,5]. It was reported that cancer cells have higher mitochondrial membrane potential (ΔΨm) than normal cells [6] Because of mitochondrial membrane potential (ΔΨm) alteration in cancer cells, delocalized cationic compounds could accumulate in the hyperpolarized mitochondria as a tumor-targeting agent for imaging and therapy [7,8,9]. For example, rhodamine-123, Acridinium, and cyanine dyes have been used for in vitro and in vivo fluorescent imaging of the altered mitochondrial membrane potential in tumor cells, and as radiotracers for nuclear imaging of tumors [6,10,11,12]. In addition, several radiolabeled triphenylphosphonium (TPP) cations were used as radiotracers for nuclear tumor imaging [13,14].

For the treatment of primary or metastatic tumors, although surgical resection is the standard treatment, the tumors are not easy to remove by surgery in many situations. Therefore, imaging-guided cancer surgery will offer more effective and safe modalities of cancer therapy [15]. When the tumor in patients are really not suitable for surgery, tumor thermal ablation is another low-invasive treatment modality [16]. Photothermal therapy (PTT) destroys cancer cells by generating heat within a tumor by absorbing specific light sources [17,18]. The heptamethine cyanine dyes also exhibits unique optical properties due to its strong absorption at NIR wavelengths, which causes photothermal effects that can trigger thermal injury and cell death both in vitro and in vivo [6,7,8,9].

Indium-111 (^111^In, half-life = 2.83 days) only emits γ-ray (23, 171 and 245 keV), and is commonly used in nuclear imaging by radiolabeling targeted molecules [19]. In this study, Indium-111 was used as a diagnostic radionuclide to label with DOTA-NIR790 for nuclear imaging of cancers. Lutetium-177 (^177^Lu, half-life = 6.73 days) is an emerging, promising medium-energy beta-emitter (490 keV) with a low-abundance gamma emission (208 keV, 11% abundance) for biomedical use. This unique property makes ^177^Lu as a suitable radionuclide candidate for both therapeutic and diagnostic purposes.

**Figure 1:**
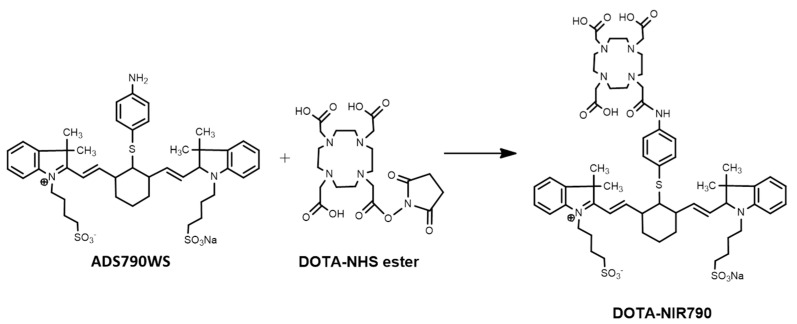
Synthesis of DOTA-NIR790.

In this study, we synthetized a cancer-targeted multimodal probe (DOTA-NIR790 as shown in Figure 1) for cancer imaging and therapy by the mitochondrial potential difference between cancers and the surrounding normal tissues. Compared with other reported cyanine-based theranostic probes, the DOTA-NIR790 has the additional chelated capabilities of radioisotope for nuclear imaging and targeted radionuclide therapy of cancers. DOTA-NIR790 is a single molecule with multi-functions for cyanine-based near-infrared fluorescence imaging and photothermal therapy of cancers, and for nuclear imaging and targeted radionuclide therapy of cancers after radioisotope-labeling. The heptamethine cyanine-based dye allowed the DOTA-NIR790 to have dual functions in cancer NIR imaging and photothermal therapy (PTT). The DOTA-NIR790 will be labeled with lutetium-177 to detect tumors by using SPECT imaging and kill cancer cells by its beta-emission. The ^177^Lu-DOTA-NIR790 enabled imaging by NIR fluorescence and by nuclear imaging (SPECT) to monitor in real-time the tumor accumulation, intra-tumoral distribution, and the situation of cancer therapy, and to guide the surgery or the photothermal therapy of the cancer.

## 2. Results

### 2.1. Characterization of DOTA-NIR790 and Radionuclide-Labeled DOTA-NIR790

The obtained crude product was purified by preparative HPLC as shown in the Supplementary Materials, Figure S1A. The collected DOTA-NIR790 (R_t_ = 9.2 min) was frozen and lyophilized overnight. Finally, the pure product was collected with a yield of 17.2%, and its chemical purity was more than 95%. The mass spectrometry and ^1^H-NMR spectrum analysis were shown in the Supplementary Materials, Figure S1B,C. As shown in Figure S2, the radiochemical purity of ^111^In-DOTA-NIR790 was more than 95% by radio-HPLC analysis, and the radiochemical purity of ^177^Lu-DOTA-NIR790 was more than 90% by ITLC.

### 2.2. Cellular Uptake of ^111^In-DOTA-NIR790

**Figure 2:**
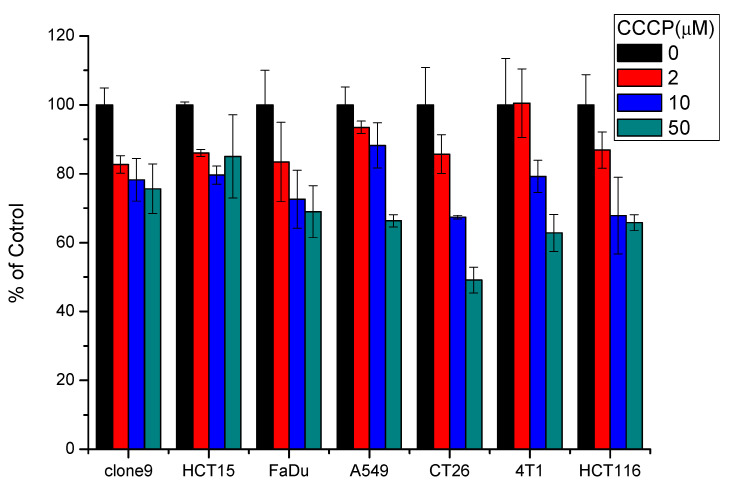
Representative uptake of ^111^In-DOTA-NIR790 in various cancer cells treated with 0, 2, 10, and 50 μM of carbonyl cyanine m-chlorophenylhydrazone (CCCP) (*n* = 3 per concentration of CCCP and cell).

In this experiment, CCCP was used to disrupt the mitochondrial potential in various cancer cells, resulting in inhibition on cellular uptake of ^111^In-DOTA-NIR790. Figure 2 illustrates representative uptake of the cancer cells treated with ^111^In-DOTA-NIR790 with/without CCCP. It is quite clear that the cellular uptake of ^111^In-DOTA-NIR790 was dose-dependently decreased by CCCP. It demonstrated that In-111-DOTA-NIR790 accumulates in cancer cells through mitochondrial membrane potential (ΔΨm) alteration.

### 2.3. Biodistribution of ^111^In-DOTA-NIR790 in Mice Bearing Subcutaneous 4T1 Tumors

**Figure 3:**
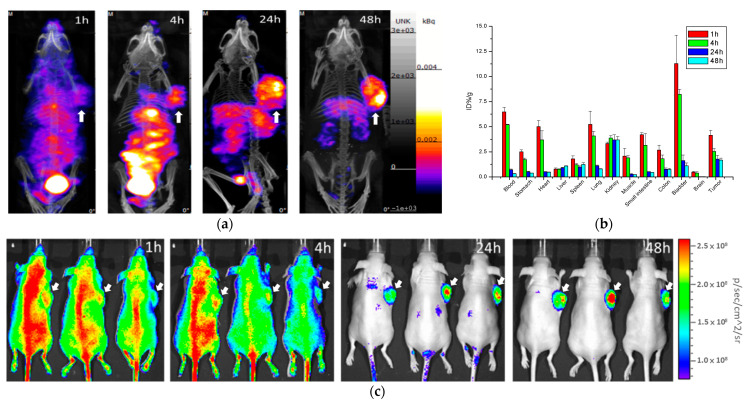
(**a**) SPECT/CT images, (**b**) biodistribution, and (**c**) NIRF images of ^111^In-DOTA-NIR790 in mice bearing subcutaneous 4T1 breast tumors (*n* = 3 at each time point). Arrow indicates tumor.

This experiment was performed by single photon computed tomography (SPECT) and near infrared fluorescence imaging (NIRF). The biodistribution of multi-functional probes ^111^In-DOTA-NIR790 in mice bearing subcutaneous tumors was evaluated. First, ^111^In-DOTA-NIR790 (~37 MBq) were intravenously injected into mice with subcutaneous 4T1 breast tumor, and imaged on a SPECT/CT at 1, 4, 24, and 48 h, as shown in Figure 3a. Then, the mice were sacrificed and the organs were collected for quantitative analysis by gamma-counter, as shown in Figure 3b. According to the biodistribution, the ^111^In-DOTA-NIR790 has a much larger accumulation amount at the tumor site than the muscle tissue with the tumor/muscle ratio of 12.84 ± 0.65 at 48 h. It is mainly due to the fact that the hydrophilic ^111^In-DOTA-NIR790 can be cleared from normal tissues and organs more quickly, which can cause ^111^In-DOTA-NIR790 to have better tumor contrast and lower side effects in future applications of therapeutic isotopes. The NIRF imaging of mice bearing tumors was received at 1, 4, 24, and 48 h after intravenous injection of ^111^In-DOTA-NIR790 by the IVIS imaging system. The results are shown in Figure 3c. The mice were sacrificed and the organs were collected for the ex vivo NIRF imaging of organs and tumor and quantitative analysis of fluorescence intensity at 48 h after injection of ^111^In-DOTA-NIR790 as shown in Supplementary materials, Figure S3.

### 2.4. Biodistribution of ^111^In-DOTA-NIR790 in Mice with Brain Metastasis of Breast Cancer

**Figure 4:**
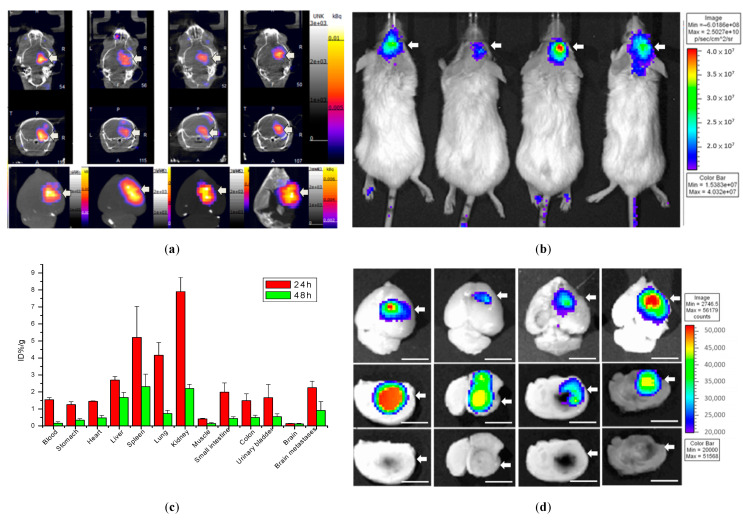
Biodistribution of ^111^In-DOTA-NIR790 in mice with brain metastasis of 4T1 breast cancer. (**a**) SPECT/CT images, (**b**) NIRF images, (**c**) biodistribution, and (**d**) ex vivo NIRF images at 48 h after administration (*n* = 4 at each time point). Arrow indicates tumor. The scale bar is 5 mm.

^111^In-DOTA-NIR790 (about 37MBq of In-111) was intravenously injected into mice with brain metastasis of 4T1 breast cancer, and the NanoSPECT/CT imaging of the brain was performed at 48 h after administration as shown in Figure 4a. In addition, the IVIS imaging system was used for near-infrared fluorescence imaging of ^111^In-DOTA-NIR790 at 48 h after administration as shown in Figure 4b. After the mice were sacrificed, their brain tissues were extracted and quantitatively analyzed by gamma-counter at 24 and 48 h after injection, as shown in Figure 4c. The ex vivo NIRF images of brains with metastatic 4T1 breast cancer were shown in Figure 4d. According to these results, this probe can specifically bind to brain metastasis of 4T1 breast tumor, and similar results are obtained in SPECT and near-infrared light imaging.

### 2.5. Biodistribution of ^111^In-DOTA-NIR790 in Mice with Subcutaneous Colon Cancer and Lung Cancer

**Figure 5:**
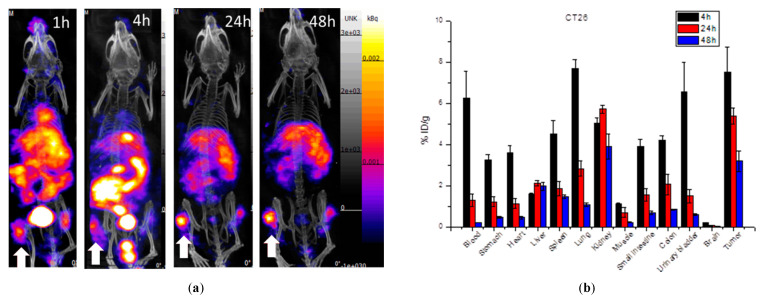
(**a**) SPECT/CT images and (**b**) biodistribution of ^111^In-DOTA-NIR790 in mice bearing subcutaneous CT-26 colon cancer (*n* = 3 at each time point). Arrow indicates tumor.

**Figure 6:**
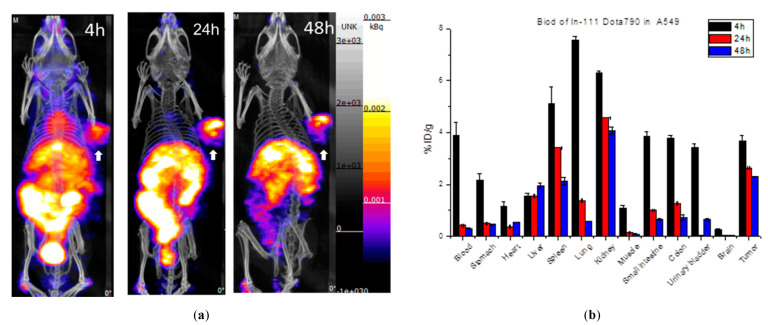
(**a**) SPECT/CT images and (**b**) biodistribution of ^111^In-DOTA-NIR790 in mice bearing subcutaneous A549 lung cancer (*n* = 3 at each time point). Arrow indicates tumor.

Figure 5 shows the single-photon computed tomography (SPECT) images and biodistribution results of the multi-function probe ^111^In-DOTA-NIR790 in mice bearing CT-26 tumor at 1, 4, 24, 48 h post-injection. The amount of tumor accumulation was 5.39 ± 0.40% and 3.19 ± 0.49% ID/g at 24 h and 48 h, respectively, and the tumor-to-muscle accumulation ratio was 15.18 ± 2.13 at 48 h. In Figure 6, the results of the SPECT images and biodistribution of ^111^In-DOTA-NIR790 in the mouse model of human lung cancer A549 showed that the amount of tumor accumulation was 2.65 ± 0.21% and 2.31 ± 0.15% ID/g at 24 and 48 h, respectively. The tumor to muscle accumulation ratio at the 48th hour was 18.98 ± 3.35. The tumor/muscle ratios in mouse model of various cancers are summarized as shown in Table 1.

### 2.6. Anti-Tumor Efficacy of ^177^Lu-DOTA-NIR790 with NIR Irradiation

**Figure 7:**
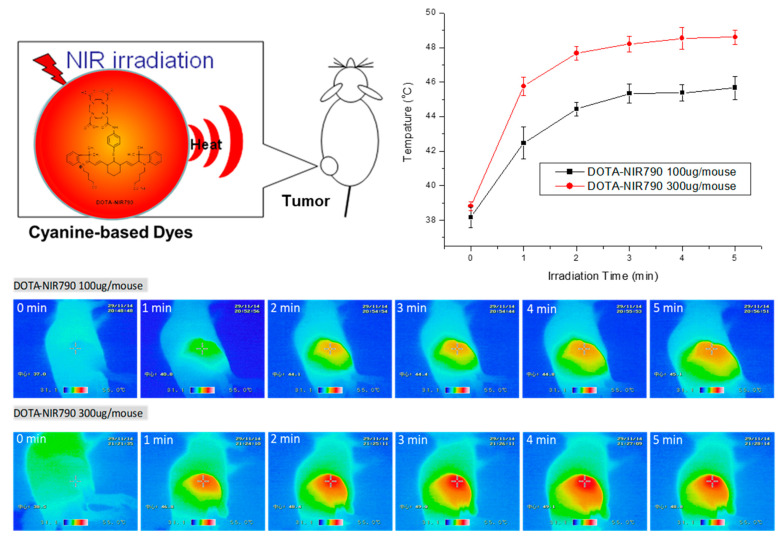
Tumor temperature measurement (*n* = 3 at each time point) and infrared thermographic map during irradiation of DOTA-NIR790 based photothermal therapy.

To evaluate the effect of DOTA-NIR790 on photothermal therapy of cancer, the results were shown in Figure 7. After exposure to laser light at 808 nm (1.8 w/cm^2^), the tumor temperature during irradiation was measured by thermal camera. The infrared thermographic map showed that the tumor temperature was effectively increased to about 48.6 °C after 5 min of irradiation, when the mice treated with 15 mg/kg of DOTA-NIR790.

**Figure 8:**
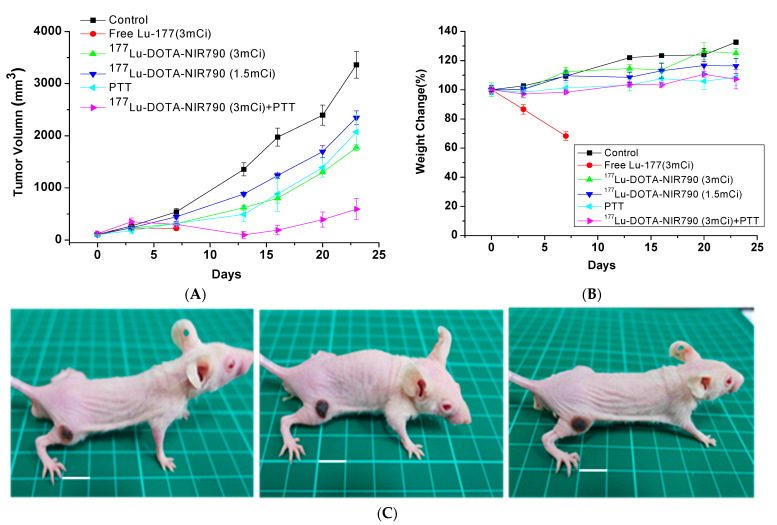
Anti-tumor effect of multi-functional tumor theranostics probes. (**A**) Tumor growth inhibition of photothermal therapy (PTT) combined with targeted radionuclide therapy by ^177^Lu-DOTA-NIR790 (*n* = 6 per group) (**B**) body weight changes, and (**C**) Representative mice treated with ^177^Lu-DOTA-NIR790 and NIR irradiation for 5 min were photographed. The scale bar is 10 mm.

As shown in Figure 8A, mice were treated with DOTA-NIR790, the tumor growth was effectively inhibited by laser irradiation, compared to the control group. The results of tumor growth inhibition also show that the tumor temperature caused by the photothermal effect of DOTA-NIR790 is elevated, which can effectively ablate the tumor and cause scarring of the tumor tissue at the illumination site. In addition, the experimental results show that photothermal therapy alone or targeted radionuclide therapy alone by ^177^Lu-DOTA-NIR790, has significant tumor growth inhibition compared with the control group. The combination of photothermal therapy and targeted radionuclide therapy by ^177^Lu-DOTA-NIR790 has an additive effect in tumor growth inhibition. Body weight loss was used as a measure of treatments-induced toxicity (Figure 8B). Representative mice treated with ^177^Lu-DOTA-NIR790 and NIR irradiation for 5 min were photographed as shown in Figure 8C. Scabs of tumor thermal ablation were observed on the flanks of mice after DOTA-NIR790-based photothermal therapy.

The body weights of both control and treatment groups were monitored throughout the experimental period. Mice treated with 111 MBq of Lu-177 lost over 30% of their weight and died at day 7. The control groups treated with PBS or targeted radionuclide therapy by ^177^Lu-DOTA-NIR790 gradually had increased their body weights by 17~30%. These values were not significantly different between each treatment, which suggested that the dose of ^177^Lu-DOTA-NIR790 was reasonably well-tolerated. Mice treated with combination of photothermal therapy and targeted radionuclide therapy by ^177^Lu-DOTA-NIR790 increased 5% of their weight at day 24. This weight change was not significantly different from other single treatment, indicating that combination of photothermal therapy and targeted radionuclide therapy mediated by ^177^Lu-DOTA-NIR790 did not result in unacceptable toxicity.

**Figure 9:**
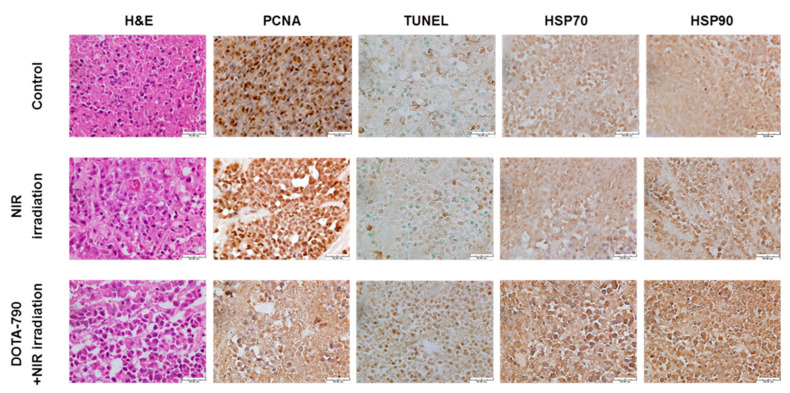
Histological and immunohistochemical analysis in 4T1 tumors treated with DOTA-NIR790-mediated photothermal therapy. The scale bar is 50 μm.

As shown in Figure 9, the histopathological and immunohistochemical analysis showed that DOTA-NIR790-mediated photothermal therapy can cause necrosis and vacuolation in the internal tissues of the tumor, inhibit the ability of tumor cell replication (decreased expression of PCNA), and the high expression of HSP protein revealed that the tumor tissue received thermal shock by the photothermal therapy.

## 3. Discussion

Globally, more than 10 million people are diagnosed with cancer every year. With a continuous rise in the number of cancer patients, the development of related drugs for cancer treatment has become an important area in the biotechnology and pharmaceutical industries worldwide. Early diagnosis and combined treatment, in particular, will be the trend for the future development of cancer treatment. The theranostic probes (DOTA-NIR790) for multimodality imaging, targeted radionuclide therapy, and photothermal therapy have been developed, as well as single-molecule probes that can be applied to tumors in various organs where drugs can accumulate in the targeted diagnostic or treated location.

This study has developed a multifunctional tumor diagnosis probe (DOTA-NIR790) that integrates near-infrared fluorescent and nuclear medical imaging, and at the same time achieves the goal of combined photothermal therapy. This study completed the synthesis, physicochemical properties, and the isotope labeling of a multifunctional tumor theranostic probe. The most suitable probe molecule was screened for DOTA-NIR790 and the establishment of its preparation method was completed. The target product DOTA-NIR790 was isolated by preparative HPLC purification, and the purity of HPLC analysis was about 95%. Mass analysis, molecular weight 1224 Da. DOTA-NIR790 was labeled with In-111 isotope and analyzed by Radio-HPLC; the radiochemical purity was about 90%. Further purification by RP-18 column could increase the radiochemical purity to more than 95%.

Radioisotope labeling of DOTA-NIR790 and SPECT imaging in the tumor-bearing animal model were performed. Near-infrared fluorescence (NIRF) imaging was performed using a non-invasive live imaging system and compared with SPECT imaging. A comparison of the results showed that ^111^In-DOTA-NIR790 began to exhibit a selective cumulative image of the tumor 24 h after administration, and showed significant tumor imaging ability by SPECT at 48 h. On the other hand, the NIRF imaging of the tumor showed that the probe could be effectively targeted to the tumor after 24 h of administration, and exhibit excellent tumor-imaging ability. The results of ^111^In-DOTA-NIR790 in the near-infrared image portion are consistent with single-photon computed tomography (SPECT). Compared with near-infrared fluorescence (NIRF) imaging, SPECT has a higher ability to detect deep tissue distribution. However, the near-infrared fluorescence (NIRF) imaging presents better tumor-targeted images in superficial tumors, which is due to the ability of the optics to penetrate the tissue.

This study also completed the tissue distribution of ^111^In-DOTA-NIR790, which showed that the probe had good tumor target. The anti-tumor effect evaluation verified that the photothermal therapy ability of the DOTA-NIR790 can effectively inhibit tumor growth. The multi-functional probe can also be labeled with the therapeutic isotope Lu-177 for targeted radionuclide therapy of cancer.

As verified by animal experiments, the application of DOTA-NIR790 in various types of cancer, such as breast cancer, colon cancer and lung cancer, shows a good tumor/muscle ratio. As shown separately in both the imaging of SPECT and NIRF, the tumor region is reflected as a thermal zone; the imaging of the tumor region presents a sharp contrast to that of non-cancerous tissue, indicating that this probe can effectively accumulate in the tumor location and generate good functional imaging. According to the previous review aimed to determine the overall sensitivity and specificity of indocyanine green (ICG) near-infrared (NIR) fluorescence in sentinel lymph node (SLN) detection in colorectal cancer (CRC) [20]. The pooled sensitivity and specificity rates were 71% and 84.6%. The ICG-NIR fluorescence is a promising technique for detecting SLNs in CRC. Although those NIR fluorescent probes do not represent sufficient specificity for cancer targeting compared with molecular targeting probes, they had been widely used in clinical for detecting sentinel lymph node (SLN) in various types of cancer, such as non-small cell lung cancer [21], gastric cancer [22], metastatic colorectal cancer [23], uterine, and cervical malignancies [24]. Therefore, the future clinical application of DOTA-NIR 790 should have cancer specificity similar to that of ICG. Furthermore, the imaging generated during the process of photothermal therapy or targeted radionuclide therapy, which was carried out to monitor the treatment condition of tumors. It was conducted by combining the probe with different radioactive doses of Lu-177, clearly showing that the size of the tumor shows a trend of diminishing as the number of days in treatment increases, compared with the control group, which indicated the DOTA-NIR790 is a potential probe for cancer diagnosis and therapy.

For functional imaging used in cancer diagnosis, single photon emission computed tomography (SPECT) used for deep-tissue tumors and near-infrared fluorescence (NIRF) imaging used for subcutaneous-tissue tumors. Both have their own advantages. This probe can combine the above two imaging methods for cancer diagnosis, depending on imaging requirements. The DOTA-NIR790 can chelate a variety of radioisotopes, such as indium-111 (In-111), lutetium-177 (Lu-177), and gallium-67 (Ga-67) for single photon emission computed tomographic imaging, gallium-68 (Ga-68) and copper-64 (Cu-64) for positron emission tomographic (PET) imaging, or even gadolinium (Gd) in non-radioactive magnetic resonance imaging (MRI) in future. The combined usage of multiple imaging techniques can increase accuracy in tumor diagnosis.

For cancer therapy, the photothermal property of cyanine-based dyes used in the probe serves to achieve the goal of tumor photothermal therapy. In addition, this probe can also apply isotopic labeling for targeted radionuclide therapy of cancer, such as lutetium-177 (Lu-177) and yttrium-90 (Y-90). In the future, the development of chemotherapy drugs with chemical bonds will serve to achieve the objective of single drugs combined with multiple treatment effects, whereby the cure rates for cancer can be improved.

## 4. Materials and Methods

### 4.1. General

ADS790WS is 2-[2-[2-(4-aminobenzenethio)-3-[(1,3-dihydro-3,3-dimethyl-1-(4-sulfobutyl)-2H-indol-2-ylidene)-ethylidene]-1-cycloxen-1-yl]-ethynyl]-3,3-dimethyl-1-(4-sulfobutyl)-3H-indolium, innersalt, monosodium, which was purchased from American Dye Source, Inc. (Montreal, QC, Canada). DOTA-NHS-ester (1,4,7,10-Tetraazacyclododecane- 1,4,7,10-tetraacetic acid mono-N-hydroxysuccinimide ester) was purchased from Macrocyclics (Dallas, TX, USA). The ESI (electrospray ionization) mass spectral data were collected on a AB Sciex 4000QTrap system (Concord, ON, Canada). ^111^InCl_3_ (indium chloride in 0.05 M HCl) was purchased from Institute of Nuclear Energy Research (INER), Taoyuan, Taiwan. ^177^LuCl_3_ was purchased from perkin elmer (Waltham, MA, USA).

Preparative reversed-phase high performance liquid chromatography (HPLC) was performed on a SHIMADZU Prominence Preparative HPLC System with a SHIMADZU SPD-20AV detector using YMC-Actus Triart C18 column (5 μm, 250 × 20 mm). Analytic reversed-phase HPLC was performed on a Waters 2695 Separations Module with a Waters 2487 Dual Wavelength Absorbance Detector plus a Bioscan radioisotope detector using YMC-Triart C18 column (5 μm, 250 × 4.6 mm). Waters Bridge column (5 μm, 200 ×4.6 mm). The flow rate was 20 mL/min for the preparative column and 1 mL/min for the analytic column running wth 60% ACN and 40% water with 0.1% TFA.

### 4.2. Synthesis of DOTA-NIR790

As shown in Figure 1, ADS790WS (83.8 mg, 100 μmol) were dissolved in 5 mL dry DMF in the presence of triethylamine (20 mg, 200 μmol). Then, DOTA-NHS (153 mg, 200 μmol) dissolved in 5 mL DMF was added, and the mixture was stirred at room temperature for 3 days. The obtained crude product was purified by preparative HPLC with a C18 reversed-phase column using 40% ACN and 60% H_2_O with 0.1% TFA as the mobile phase. The peak containing the desired product was collected (Rt = 9.2 min), and the collected solution was frozen and lyophilized overnight. ESI-MS: m/z = 1224.5 for [M + H]^+^ (1224.3 calcd for [C60H79N7NaO13S3]^+^).

### 4.3. Preparation of ^111^In-DOTA-NIR790 and ^177^Lu-DOTA-NIR790

^111^InCl_3_ (370 MBq) [^111^InCl3; Institute of Nuclear Energy Research (INER), Taoyuan, Taiwan, 16430 MBq/mL in 0.05N HCl; pH 1.5–1.9] or ^177^LuCl_3_ (370 MBq) were added to DOTA-NIR790 (10μg) diluted in 300 μL of 0.2 M sodium acetate buffer (pH 5.5), respectively. The reaction mixture was incubated for 1 h at 37 °C with constant shaking. The primary products were purified by RP-18 column using phosphate-buffered saline (PBS) as the washing buffer and ethanol as elution buffer. The radiochemical purity of ^111^In-DOTA-NIR790 was evaluated by radio-HPLC analysis. The radiochemical purity of ^177^Lu-DOTA-NIR790 was evaluated by the instant thin layer chromatography (ITLC) with 0.1M Na-citrate (pH 5.0) as the solvent (^177^LuCl_3_: Rf = 0.9~1.0, precursor-bound ^177^Lu: Rf = 0~0.1).

### 4.4. Cellular Uptake of DOTA-NIR790

For cellular uptake, clone9, HCT-15, FaDu, A549, CT26, 4T1, and HCT-116 cells were seeded in 24-well plates at a density of 10^5^ cells per well 24 h before the assays. The medium was removed, and the 3.7 MBq of ^111^In-DOTA-NIR790 in 0.5 mL of medium containing 0, 2, 10, or 50 μM of carbonyl cyanine m-chlorophenylhydrazone (CCCP) were added per well (*n* = 3 per concentration of CCCP and cell). After 24 h incubation, the medium was removed, and the cells were washed twice with 1 mL of PBS and lysed by the addition of 0.2 mL of 0.1 M NaOH. Cell lysates were collected, and the radioactivity was measured by a γ-counter. The cellular uptake values were normalized to that of CCCP-untreated cells, and the normalized intensity (%) is calculated as the percentage of radioactivity obtained by CCCP-treated and CCCP-untreated cells.

### 4.5. Animal Model

Subcutaneous tumor model. Female nude mice that were 5 to 6 weeks old were purchased from the National Laboratory Animal Center (Taipei, Taiwan). The mice were housed with a 12-h light/dark cycle and allowed free access to water and standard diet. The cancer cells (1 × 10^6^) were inoculated subcutaneously on both right and left flanks of 5 to 6 weeks old female nude mice, respectively. Tumor sizes and body weights were measured every three days for the duration of the experiment. Tumor volume was calculated as π/6ab^2^ where a is the length of the tumor, and b is the width of the tumor.

Brain metastasis model. Murine 4T1-luc2 breast cancer cells (ATCC^®^ CRL-2539™) expressing firefly luciferase (luc2 vector) were cultured in Dulbecco’s Modified Eagle’s Medium (DMEM) supplemented with 10% heat-inactivated fetal bovine serum, penicillin (100 U/mL)/streptomycin (100 μg/mL) in 10 cm tissue culture plates in a 5% CO_2_-containing incubator at 37 °C. Eight-week-old female BALB/c mice were used in this study (*n* = 4 at each time point). During the tumor implantation, the mice were anesthetized by exposure to 1% to 3% isoflurane. A total of 2 × 10^4^ of 4T1-luc2 tumor cells suspended in 2 μL of phosphate buffered saline (PBS) were slowly injected into the right caudate putamen 0.5 mm anterior and 2.0 mm lateral to the bregma at a depth of 3 mm from the dura over a 3-min duration. The needle was left in place for 5 min and then withdrawn slowly. The scalp wound was closed with 6-0 polydioxanone suture.

### 4.6. Biodistribution of Radioisotope-DOTA-NIR790

When the tumors reached a volume of 150 to 200 mm^3^ in the subcutaneous tumor model, mice received an intravenous injection of ^111^In-DOTA-NIR790 (equivalent to 10 MBq of ^111^In) or ^177^Lu-DOTA-NIR790 (equivalent to 37 MBq of ^177^Lu) (*n* = 3 at each time point). In brain metastasis model, the mice received an intravenous injection of ^111^In-DOTA-NIR790 at 10 days after tumor implantation. The distribution of ^111^In-DOTA-NIR790 or ^177^Lu-DOTA-NIR790 in the mice bearing tumors was evaluated by NanoSPECT/CT (Bioscan Inc., Washington, DC, USA) at 1, 4, 24, and 48 h after intravenous injection. The mice were sacrificed by cervical vertebra dislocation at 48 h after administration. The plasma, tumor, and normal tissue were collected, and the uptake of radioactivity was measured by a gamma counter. The distribution data obtained using radioactivity count methods are plotted as %ID/g.

### 4.7. Near-Infrared Fluorescence (NIRF) Imaging of ^111^In-DOTA-NIR790

The NIRF imaging of mice bearing tumors was studied at 1, 4, 24, 48, and 96 h after intravenous injection of ^111^In-DOTA-NIR790 (equivalent to 10 MBq of ^111^In) by the IVIS imaging system (Xenogen, Alameda, CA, USA) (*n* = 3 at each time point). The mice were anesthetized with a mixture of oxygen and isoflurane, and were placed on a 37 °C animal plate. The near-infrared fluorescence (NIRF) data were collected with a two second exposure time and an ICG filter set with excitation at 710–760 nm and emission at 810–875 nm. Quantitative analysis of fluorescence intensity in the images was performed using the region-of interest (ROI) function of the Living Image^®^ software (Caliper Life Sciences Inc., Hopkinton, MA, USA).

### 4.8. Anti-Tumor Efficacy of ^177^Lu-DOTA-NIR790 with NIR Irradiation

After the subcutaneous tumors reached a volume of 150 to 200 mm^3^, mice received an intravenous injection of DOTA-NIR790 or ^177^Lu-DOTA-NIR790 (equivalent to 55.5 and 111 MBq of ^177^Lu). All mice were randomly divided into several groups (*n* = 6 per group), including normal saline, DOTA-NIR790 based photothermal therapy, radiotherapies with 55.5 and 111 MBq of ^177^Lu-DOTA-NIR790, and combination of radiotherapy and photothermal therapy, respectively. For photothermal therapy, 15 mg/kg of DOTA-NIR790 were administered via tail vein injections, and the tumors were exposed to the NIR laser with a spot size of 5mm at 1.8 W/cm^2^ for 5min at 24 h after administration. The size of subcutaneous tumors and body weight change of mice were recorded.

### 4.9. Immunohistochemical Analysis of Tumors with DOTA-NIR790-Mediated Photothermal Therapy

To evaluate the treatment effect, the mice with subcutaneous tumors were sacrificed, and tumor tissues were collected, after treatments of PBS (control), NIR irradiation alone, or DOTA-NIR790-mediated photothermal therapy. For the histopathological and immunohistochemical analysis, the section slides were stained with hematoxylin and eosin (H&E), proliferating cell nuclear antigen (PCNA), terminal deoxynucleotidyl transferase dUTP nick end labeling (TUNEL), and Heat-shock proteins (HSPs) as described in previous reports [25,26,27].

## Figures and Tables

**Table 1:** Tumor/Muscle Ratio of ^111^In-DOTA-NIR790 in various cancer at 48 h after administration.

Cancer Model	Tumor/Muscle Ratio
4T1 breast cancer	12.84 ± 0.65
CT26 colon cancer	15.18 ± 2.13
A549 lung cancer	18.98 ± 3.35

## Data Availability

The data presented in this study are contained within the article.

## Supplementary Materials

The following are available online at https://www.mdpi.com/article/10.3390/ijms222212214/s1.
