# Supplementary material for: Multifunctional Cyanine-Based Theranostic Probe for Cancer Imaging and Therapy
Source: Int J Mol Sci. 2021 Nov 11;22(22):12214. doi: 10.3390/ijms222212214 (PMC8625430; doi:10.3390/ijms222212214)
Supplement: Supplementary file 1 [file ijms-22-12214-s001.zip › ijms-1430875-supplementary.pdf]

Supplementary Materials

Multifunctional Cyanine-Based Theranostic Probe for Cancer Imaging and Therapy

Cheng-Liang Peng [1,*], Ying-Hsia Shih [1], Ping-Fang Chiang [1],
Chun-Tang Chen [1] and Ming-Cheng Chang [1]

[1] Isotope Application Division, Institute of Nuclear Energy Research, P.O. Box 3-27, Longtan, Taoyuan 32526, Taiwan; ShihY@iner.gov.tw (Y.-H.S.); ckdopamine@iner.gov.tw (P.-F.C.); ctchen@iner.gov.tw (C.-T.C.); mcchang@iner.gov.tw (M.-C.C.)

* Correspondence: clpeng@iner.gov.tw; Tel.: +886-3-4711400 (ext. 7298); Fax: +886-3-4711416

A.

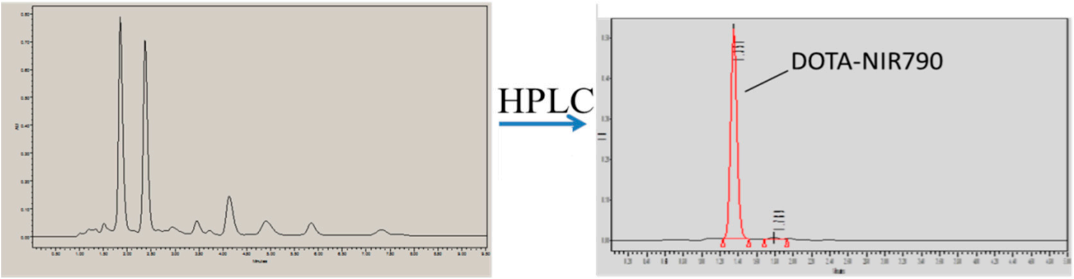

B.

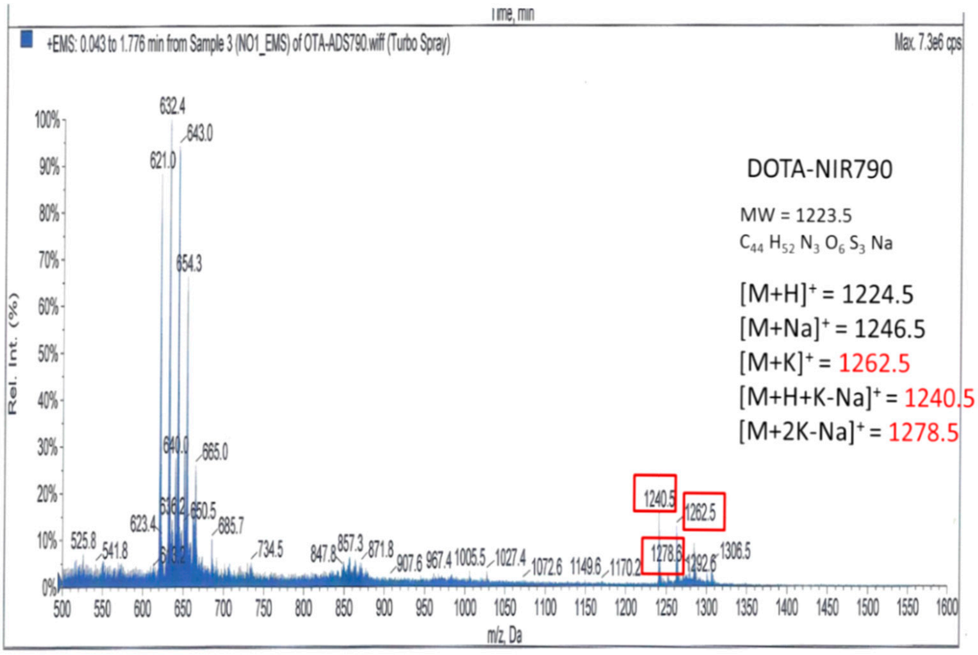

C

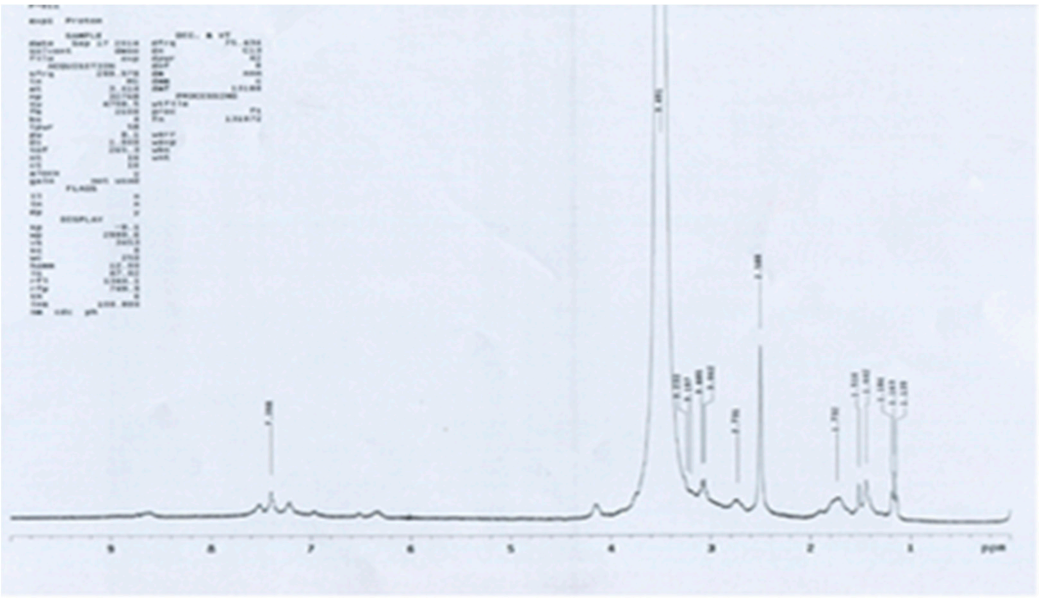

Figure S1. (A) Purification, (B) mass spectrometry, and (C) ^{1}H-NMR spectrum analysis of the multifunctional tumor imaging probe molecule (DOTA-NIR790).

(A)

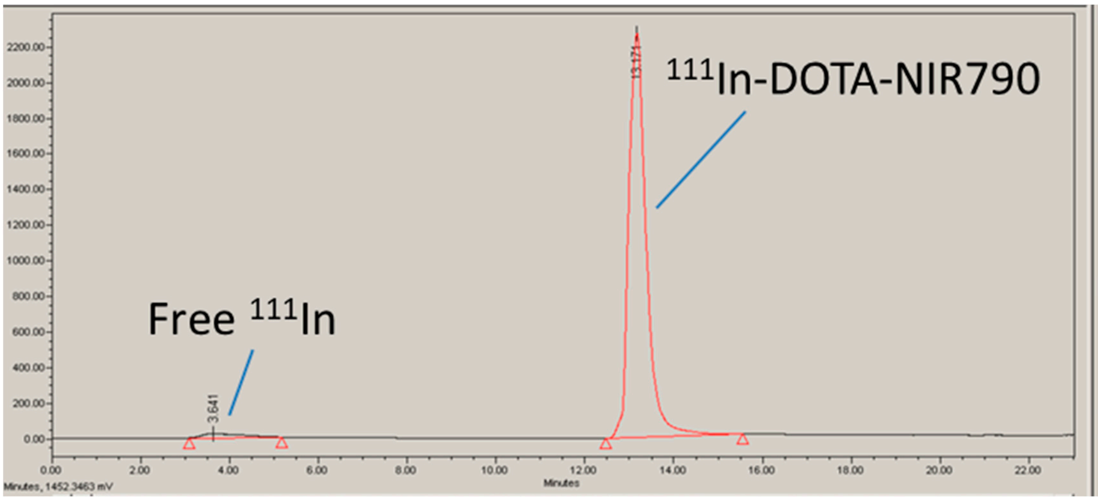

(B)

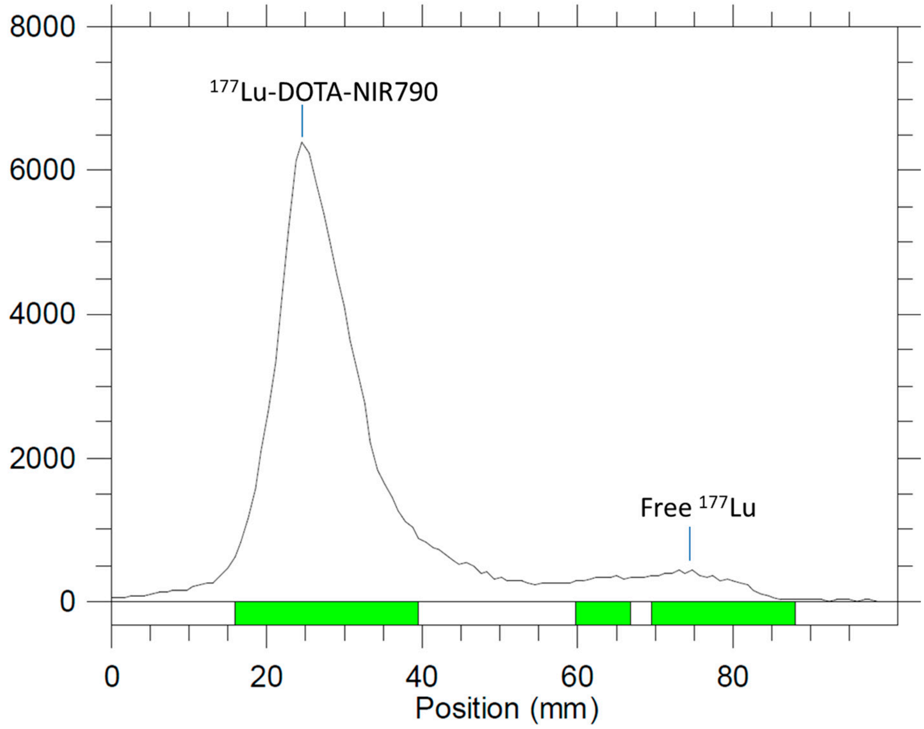

Figure S2. (A) Radiochemical purity of ^{111}In-DOTA-NIR790 by Radio-HPLC. (B) Radiochemical purity of ^{177}Lu-DOTA-NIR790 by ITLC.

(A)

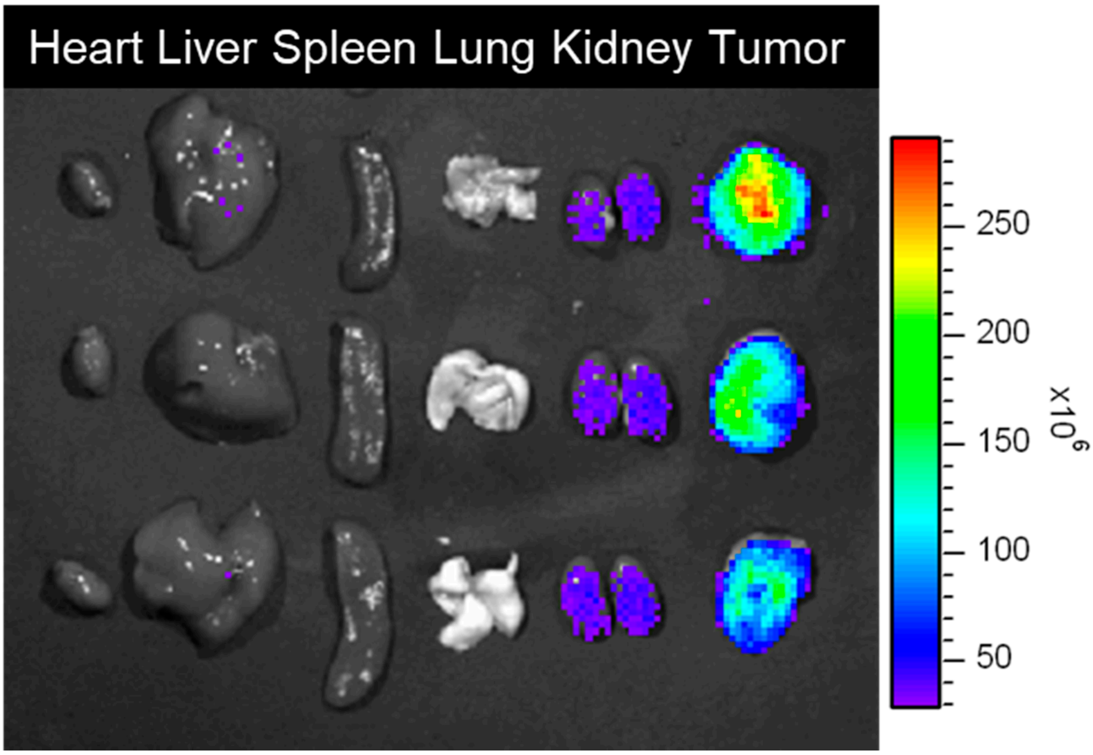

(B)

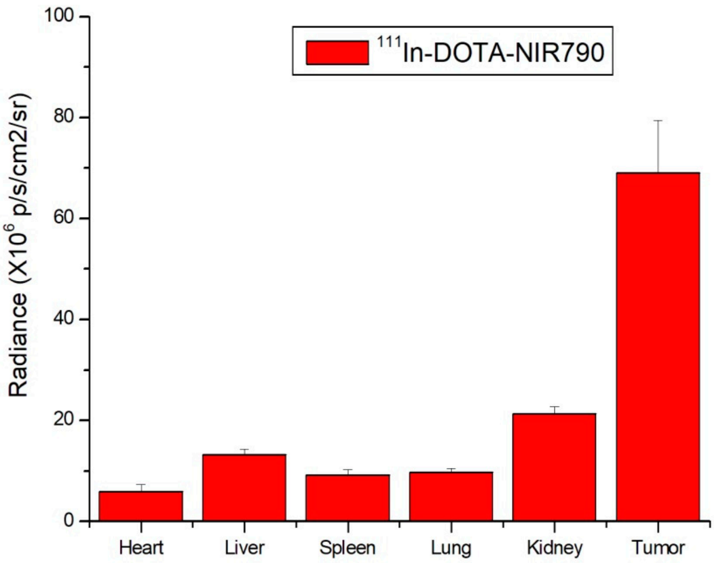

Figure S3. (A) *Ex vivo* NIRF images of organs and tumor harvested at 48h after injection of ^{111}In-DOTA-NIR790. (B) Quantitative analysis of fluorescence intensity in the *ex vivo* NIRF images.
